# Processing and extraction methods of medicinal cannabis: a narrative review

**DOI:** 10.1186/s42238-021-00087-9

**Published:** 2021-07-19

**Authors:** Masoumeh Pourseyed Lazarjani, Owen Young, Lidya Kebede, Ali Seyfoddin

**Affiliations:** 1 Drug Delivery Research Group, School of Science, Faculty of Health and Environmental Sciences, Auckland University of Technology, Auckland, New Zealand; 2 School of Science, Faculty of Health and Environmental Sciences, Auckland University of Technology, Auckland, New Zealand

**Keywords:** Extraction, Cannabinoids, Terpenes, Drying, Solvents, Processing, Cannabis

## Abstract

**Introduction:**

As the cannabis industry transitions from a black market to a legal market, product development, and methods of extraction have become a focal point. To date, more than thousands of chemical constituents have been identified from the cannabis plant, all of which possess different chemical properties that require different conditions for preservation during drying and extraction. However, scientific publications that explore these areas for the cannabis plant are currently lacking.

**Method:**

This is a narrative review paper which focuses on critiquing drying and extraction methods of *Cannabis sativa* L. plant. Relevant keywords such as medicinal cannabis, extraction, solvent, cannabinoids, and terpenes have been searched in PubMed, EMBASE, MEDLINE, Google Scholar, and Cochrane Library (Wiley) databases.

**Result:**

To find relevant papers for this narrative review, 93 papers have been reviewed. Among them, 12 irrelevant papers were discarded. The excluded papers were either about hemp seed oil or hemp fiber and protein. Based on this review, solvent extraction is the most common method for cannabis plants. Although solventless and hydrodynamic extraction are known for their high yield and feasibility, more investigation is needed in these areas. Regarding the drying process, hang-drying is the most convenient method; however, it may be substituted by freeze-drying in the near future.

**Discussion:**

This review analyses various drying and extraction processes to guide the selection of suitable methods for various types of cannabis products and applications. This is done by outlining traditional and modern methods of drying techniques, exploring the importance of solvents for extraction, visiting solventless extraction procedures, and finally comparing conventional and alternative methods of extraction.

**Conclusion:**

In conclusion, based on the current knowledge, using organic solvents is the most convenient method for medicinal cannabis extraction. However, more research is needed for some of the drying and extraction methods. Also, developing a green and sustainable cannabis extraction method should be considered for future studies.

## Introduction

Cannabis is a flowering plant from the Cannabaceae family and genus *Cannabis*. *Cannabis sativa* and *Cannabis indica* are generally well known, while subspecies *Cannabis ruderalis* is often overlooked due to its limited ability in producing active compounds (Gloss 2015). Hybrid species are variable depending on the parent plant; they can be *sativa* dominant, *indica* dominant, or balanced. Within the genus, the number of species is disputed, and the traditional nomenclature of *sativa* and *indica* may not be correct or useful in determining therapeutic potential. In any case, cannabis is dioicous, meaning it exhibits both male and female reproductive structures in separate individual plants. Female cannabis plants produce more glandular trichomes compared to the male plant. Among all the known compounds in the cannabis plant, cannabinoids and terpenes are the most active compounds with therapeutic potential which largely synthesized in those glandular trichomes. These compounds have shown to have therapeutic effects on a range of conditions such as metabolic disorders, neurodegenerative disorders, movement disorders, anorexia in HIV patients, nausea, and pain after chemotherapy in cancer patients (Namdar et al. 2018; Romano and Hazekamp 2013) (Table 1).

**Table 1:** Common cannabinoids and their molecular formulas

Cannabinoid name	Usual abbreviation	Molar mass (g mol^−1^)	Molecular formula
(-)-trans-Δ^9^-tetrahydrocannabinol	∆^9^-THC	314.472	C_21_H_30_O_2_
(-)-trans-Δ^8^-tetrahydrocannabinol	∆^8^-THC	314.472	C_21_H_30_O_2_
(-)-trans-Δ^9^-tetrahydrocannabinolic acid A	THCA	358.482	C_22_H_30_O_4_
Cannabidiol	CBD	314.472	C_21_H_30_O_2_
Cannabidiolic acid	CBDA	358.482	C_22_H_30_O_4_
Cannabinol	CBN	310.440	C_21_H_26_O_2_
Cannabinolic acid	CBNA	354.450	C_22_H_26_O_4_
Cannabigerol	CBG	316.488	C_21_H_32_O_2_
Cannabigerolic acid	CBGA	360.498	C_22_H_32_O_4_

As the cannabis industry transitions from a black market to a legal market, product development, and methods of extraction have become a focal point. Traditionally, the dried cannabis flower has been a popular product for the use of smoking and vaping. However, as the industry expands, the need for cannabis products in different forms and higher potency also increases. Currently available products, medicinal or recreational, come in the forms of topicals, edibles, beverages, and vaporization cartridges. Each product type presents its own set of advantages and disadvantages allowing for customization to serve a particular purpose (Blake and Nahtigal 2019). For pharmaceutical and food applications, the extraction and isolation of active components and combinations of identified cannabinoids are critical steps that should be explored (Fathordoobady et al. 2019).

The separation of bioactive compounds has recently become rapidly sought after by the pharmaceutical and food industries. This is due to the increased understanding of the dynamic nature and potential of diverse bioactive molecules from natural sources (Azmir et al. 2013). To further continue scientific research on the selection, identification, and characterization of bioactive compounds, the selection of a suitable extraction process is imperative (Azmir et al. 2013). Failing to designate a fitting method of sample preparation can jeopardize any analytical procedure resulting in unfavorable outcomes. However, the field of extraction is often neglected and is not studied as thoroughly as other processes. This creates a gap in the literature that should be explored more extensively (Smith 2003). The process of extraction is commonly employed to obtain target bioactive compounds from complex plant matter, yet it can also be altered to cater for many purposes, for instance, increasing the selectivity and sensitivity of bioassays by increasing the concentration of a target compound, as well as providing a potent and reproducible sample matrix (Smith 2003). Valizadehderakhshan et al. (2021) compared different extraction methods for seed and trichomes in *Cannabis sativa* L. They also reviewed various parameters that affect cannabinoid transformation after extraction (Valizadehderakhshan et al. 2021).

Different methods of extraction will yield varying degrees of extract quality and composition depending on the procedure and substances used (Blake and Nahtigal 2019). This review focuses on various drying and extraction methods while comparing conventional and most recent methods. For example, conventional methods of extraction including Soxhlet and dynamic maceration have longer extraction time and large amounts of solvent are required to complete the extraction process (Agarwal et al. 2018). Recent methods including ultrasonic-assisted, microwave-assisted, supercritical fluid, and pressurized liquid extraction processes can be considered as an alternative, slightly greener, options as opposed to the conventional methods. These procedures reduce the need for synthetic and organic solvents, cut down on operational time, and produce a better quality extract with a higher yield (Azmir et al. 2013). Solventless methods such as dry sieve and water extraction are particularly known to extract entire trichomes. Hydrocarbon extraction methods can be used to avoid unwanted water and pigments such as chlorophyll. Ethanol can extract flavonoids, while carbon dioxide can be manipulated to extract different compounds depending on the conditions (Blake and Nahtigal 2019).

The characteristics of the product must be considered when deciding on a method. For example, depending on the application, cannabinoids can be extracted in either acidic or neutral form. The preservation of acidic cannabinoids requires extraction to be completed at room temperature (Citti et al. 2016). To decarboxylate acidic cannabinoids into neutral form, high temperatures are recommended for extraction, although a higher temperature may result in the loss of some terpenes and minor constituents (Fathordoobady et al. 2019). Therefore, the selection of an appropriate extraction procedure will benefit future stages of development by minimizing the requirements for refinements (Blake and Nahtigal 2019). To further understand the processes and possible outcomes, this review will explore different methods of drying and extraction procedures used for the cannabis plant.

## Method

This paper is a narrative review paper which focuses on drying, extraction, and post-extraction methods for *Cannabis sativa* L. plant. A combination of keywords such as medicinal cannabis, extraction, solvent, and cannabinoids have been searched in databases such as PubMed, EMBASE, MEDLINE, Google Scholar, and Cochrane Library (Wiley) from 1977 to 2021 in English.

## Results

The focus of this narrative review was on *Cannabis sativa*, initially where 93 papers were identified. Papers on various drying and extraction methods specifically for *Cannabis sativa* L. were included while those for using hemp as fiber and protein sources were excluded. Overall, 12 papers about cannabis seed oil, hemp seed oil, or hemp plant were excluded as this review focuses on the oil coming from flowers. In the end, 81 related papers about various drying, extraction, and post-harvest processes were carefully reviewed.

### Influence of external factors on cannabis

External factors such as light duration, oxygen, and harvest time (floral maturity) have been shown to influence the secondary metabolite production in cannabis (Liu et al. 2015; Namdar et al. 2019). A 4-year study by Lindholst (2010) found that cannabinoid stability is affected by temperature, light, and air. Three conditions were used to store cannabis resin (hashish slabs) and extract (by the solvent): room temperature and 4 °C both with visible light exposure and darkness, and − 20 °C in darkness. The study identified that in cannabis resin, light exposure can affect the decarboxylation of THCA and the degradation of THC. This is evident as the half-life increased by 40% in darkness. However, it was observed that light was only partially influential. The resin samples that were placed at room temperature, in either light or dark settings, only exhibited little differences in the degradation of neutral THC. The dense color and structure of resin are thought to be the reason behind the reduced light sensitivity of THC. Accordingly, it is suspected that the exposure of light on resin only reaches the cannabinoids on the surface resulting in low degradation levels. This theory is further illustrated when a comparison was done between the degradation levels of both acidic and neutral THC levels in cannabis resin and cannabis extract. It was observed that both the neutral and acidic forms of THC in the cannabis extract degraded significantly more through light exposure. Furthermore, compared to resin, cannabis extract had a 10 times lower half-life (35 days for extract and 330 days for resin), while THCA decreased to nondetectable levels after 140 days. The neutral forms, in the extract, increased during this period, although THC concentrations were reduced to 1.7% after 2 years at room temperature with light exposure. It was also found that extracts stored at 4 °C showed the same pattern, but degradation was slower, while at − 20 °C all measured cannabinoids remained unchanged during the study period (Lindholst 2010). Danziger and Bernstein (2021a, b) evaluated the effect of light on three chemovars of cannabis under four different light conditions. In this study, light as the key factor affected the profile and yield of cannabis chemovars. To be precise, using blue to red lights (1:1 and 1:4 ratios) had the highest yield compared to white LED light. In addition, CBGA as a primary cannabinoid and precursor for many cannabinoids increased by using blue light (Danziger and Bernstein 2021a). The same authors in another study investigated the effect of architectural manipulation of the plant on the cannabinoid’s standardization. Defoliation, removing primary and secondary branches, and pruning have been considered as a part of eight various architectural manipulation treatments in different light intensities. Results showed that plant architectural modulation affects cannabinoid profile while no changes has been reported in the decarboxylation of cannabinoids (Danziger and Bernstein 2021b). Saloner and Bernstein (2021) evaluated the effect of nitrogen supply as an environmental factor on cannabinoids and terpenes. Results showed that the concentration of THCA and CBDA decreases by increasing the amount of nitrogen 69% and 63%, respectively. Bernstein et al. (2019) evaluated the effect of common minerals on the cannabinoid profile by adding humic acid (HA), phosphor (P), nitrogen (N), and potassium (K) to the commercial treatment into irrigation solution for a high THC cannabis chemovar. Each of the supplements affected the cannabinoid concentrations differently based on the organ and its location in the plant. For example, adding NPK supplement increased 71% the amount of CBG in the flower, while it decreased the amount of CBN in the flowers and leaves by 38% and 36%, respectively (Bernstein et al. 2019).

### Drying

For many applications, the dried version of the cannabis herb is required; however, like many plants, cannabis contains approximately 80% water. For this reason, drying is considered an essential step for product development (Hawes and Cohen 2015). Drying the plant not only prevents the growth of microorganisms that would otherwise rot plant tissue (based on ASTM D8196-18 which is a standard practice for determination of water activity (aw) in cannabis flower), it would also enable long term storage while maintaining potency, taste, medicinal properties, and efficacy (Hawes and Cohen 2015). This is done by maintaining the water activity level between 0.55 and 0.65 aw, minimizing the risk of mold or fungal infection while preserving the quality of the flower (ASTM D8196-18).

### Air-drying, also known as hang-drying

**Fig. 1:**
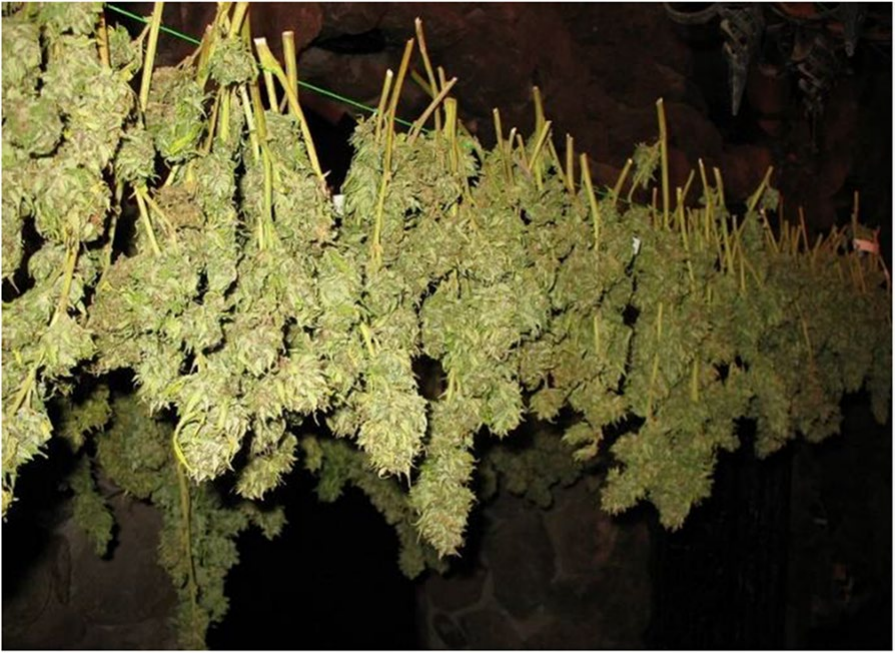
Air-drying (hang-drying) of the cannabis plant

Hang-drying or air-drying is considered the oldest way of drying cannabis plants after harvest (Fig. 1) that requires no dedicated equipment (Ross and ElSohly 1996). Slow-drying includes placing whole plants or separated inflorescence in a cool dark room with a temperature between 18 and 25 °C and humidity between 45 and 55%, either hung from a string or laid out on drying screens (Hawes and Cohen^ 2015^). Ross and ElSohly (1996) applied four treatments for air-drying to evaluate the efficacy of each condition in producing the highest yield of cannabis products. The treatments were extracted immediately, after the flower harvest at room temperature (0.29% yield, w/v) (A), after 1 week of air-drying at room temperature (0.20% yield based on wet material, v/w) (B), after 1 week of air-drying followed by storage for 1 month at room temperature (0.16% yield based on wet material, w/v) (C), and air-drying for 1 week and stored in paper bags for 3 months at room temperature (0.13% yield based on wet material, v/w) (D). From this experiment, it was found that the yield from treatments A to D decreased from 29 to 13%, respectively (Ross and ElSohly 1996). Inconveniences of this method include the manual removal of leaves and buds from the stem as well as the time taken to complete the overall process. The separation is crucial as different parts dry at different rates; therefore, a lack of completing this step may result in uneven drying. Consequently, a disadvantage of removing buds from stems is the possibility of producing a product with a harsher taste. Another detriment of this method is the involvement of gravity. The water from the top part of the plant will absorb into the lower parts leading to a slower and uneven drying process. To speed up the procedure, heaters, fans, and dehumidifiers can be used. However, fast-drying can lead to a harsher taste as opposed to slow-drying which produces smoother tasting products. It is also believed that speeding up the drying process can prevent the plant from reaching peak potency in the curing phase (Hawes and Cohen 2015). Coffman and Gentner (1974) evaluated the effect of drying conditions on the cannabinoid profile. They stored the cannabis hang dried leaves in 65, 85, and 105 °C for 1, 4, 16, and 64 h to compare the mean percentage of total cannabinoid content. The results were shown that the percentage of total cannabinoids was decreased by increasing time and temperature. To be precise, the percentage mean weight loss of total cannabinoids increased from 7.5 to 11% in 65 °C after 1 h and 105 °C after 64 h, respectively.

### Oven-drying

A faster direct method of drying is the oven-drying approach (Mujumdar 2006). This method can be carried out in either a vacuum chamber, vacuum desiccator, or in a drying oven with or without air circulation (Hawes and Cohen 2015). To illustrate the outcomes of the process, an early study tested out four different oven conditions to compare the end products. Inflorescences were dried for 1, 4, 16, and 64 h at 65, 85, and 105 °C. After extraction with ethanol, gas chromatography showed that the yield of CBD and THC decreased as the temperature and time of drying increased. It was also observed that at temperature 105 °C, the thermal degradation of THC increased the CBN content (Coffman and Gentner 1974). CBN is considered a less potent psychoactive and mild analgesic; therefore, conversion of THC to CBN will decrease the therapeutic potential (Citti et al. 2016).

Additionally, using high temperatures and excessive drying can result in the loss of key components (Hawes and Cohen 2015). This statement can be the reason for the lack of information about using oven dying in the cannabis industry. This was highlighted in a study that compared the ratio of cannabinoid and by-product produced during vaporization. The cannabis material was placed in the desiccator for 5 days to dry out, while the smoke condensate and vaporized condensate trapped in the organic solvent were dried with a rotary evaporator at 40 °C. These approaches had produced intense fragrance which is indicative of the loss of terpenoids and other volatile components (Pomahacova et al. 2009).

### Freeze-drying

Freeze-drying (also known as lyophilization) has become a popular option due to the increasing demand for high-quality medicinal cannabis. The freeze-drying method holds the cannabis plant at temperatures far below those of air or oven, while removing the water content, in the form of vapor, via sublimation in a vacuum chamber (Mujumdar 2006). The nascent legal cannabis industry claims that freeze-drying preserves the volatile compounds and acidic form of cannabinoids (Tambunan et al. 2001). It is generally agreed that the end products of freeze-drying are considered high quality compared to other methods of drying. This is due to the structural rigidity found on the surface of frozen materials where sublimation occurs, preventing the disintegration of the solid matrix and resulting in a porous, unaltered structure (Mujumdar 2006). When assessing the end product produced by freeze-drying, it was found that the composition is largely unaffected from that found in the plant (Tambunan et al. 2001). A disadvantage of freeze-drying is the cost of operation. This procedure requires an intense amount of energy to maintain such temperatures, vacuum, and long-running time (Mujumdar 2006).

Comparing the different drying methods, we can safely state that the approach elected will affect the yield and cannabinoid profiles in the extracts. Therefore, the selection of a drying procedure will largely alter the outcomes (Coffman and Gentner 1974). The process of hang-drying cannabis was found to be time-consuming as it can take several days, while the main factors that increase the rate of drying were determined to be moving air and low humidity (Ross and ElSohly 1996). In contrast, the oven-drying method was observed to be faster, but readily volatile compounds and neutral forms of cannabinoids decreased in extracts to almost non-detectable concentrations, affecting therapeutic potential (Coffman and Gentner 1974). To address this issue, freeze-drying is thought to be the preferred method. Freeze-drying enables the preservation of flavor qualities in many foods, themselves often due to the presence of volatile compounds (Tambunan et al. 2001).

In all the drying methods mentioned above, humidity, temperature, ventilation rate, and time are the most important parameters to be optimized. Incorrect drying conditions may cause decarboxylation of acidic cannabinoids and loss of terpenes. The presence of light, oxygen, and heat may also cause degradation in cannabinoids and terpenes and can affect the taste (Jin and Chen 2019).

### Curing

Curing is the final post-harvest procedure that allows for the development of the maximum flavor in the cannabis plant (Vogel 2018). Jin et al. (2019) believed that the best temperature and humidity for curing are at 18 °C and 60% RH for 14 days. Green et al. (2018) suggested keeping the trimmed flowers in a can for up to 4 weeks in a dark cupboard while opening the lid every day for about 6 h is the best method for curing (Jin and Chen 2019). At temperatures between 15–21 °C and 45–55% humidity, enzymes and aerobic bacteria will be in the optimum condition to breakdown undesired sugars and degrade minerals. Curing can reduce the harsh smell and the sense of throat burning during smoking or vaping as well as increasing the shelf life by minimizing mold growth. It is also believed that curing can increase cannabis potency as the number of cannabinoids such as THC and CBN will increase by curing. Although curing is one of the most significant post-harvest stages for the cannabis plant, there are not enough academic investigations around this area.

### Extraction methods

Cannabis extraction can be used to concentrate target components for product development. There are important parameters that can affect the yield of the cannabis extract such as mean particle size, size distribution, temperature, rate of agitation, and extraction time (Fathordoobady et al. 2019). Solventless, solvent-based, convention, and alternative methods of extraction are explored concerning cannabis extraction.

### Solventless extraction

Long-established solventless methods such as dry-sieving, water extraction, and rosin press extraction lack coverage in literature due to outdated techniques and difficulty in scaling despite having simple procedures. Dry sieve extraction produces a powder-like Kief with a potency of approximately 35–50% THC. The process of dry-sieving begins by beating dried cannabis against a mesh screen and forcing the trichomes to separate and fall off. The final product can either be pressed further into hashish or mixed with dried flowers. This simple procedure is time-consuming and labor-intensive, therefore, not popular for the industrial level. Water extraction produces roughly the same potency of THC as the dry sieve method, although it also depends on the potency of the starting material. The procedure begins by placing the cannabis plant in a mesh bag immersing it in ice water and finally stirring it to knock the trichome off. The trichome is further filtered through a series of screens then allowed to settle before collecting and drying the final product, commonly known as water hash or bubble hash. Similarly, to dry sieving, this process is difficult to upscale as well as limited control of potency (Blake and Nahtigal^ 2019^).

Solventless extraction exploits the fact that cannabinoids are semi-liquid and can be extracted by suitable heating and pressure. Rosin extraction uses compression and heat to obtain oils and rosin. Rosin extraction can be as simple as using a hair straightener for recreational extractions. For more commercial medicinal applications, a modified hat press is adopted. For both methods, high pressure at low temperatures is not achievable; therefore, the retention of terpenes is limited (analytical cannabis.com) (Lamy et al. 2018). To prevent high-temperature changes, a typical pneumatic press can be used, exerting some lower temperatures and preserving the terpenes. Pressures up to 137.8 MPa can be generated in some pneumatic presses.

### Solvent-based extraction

Solvent-based extraction methods such as Soxhlet, maceration both static and dynamic, ultrasonic-assisted extraction, and microwave-assisted extraction require a solvent to complete the extraction process. A variety of solvents can be used to extract cannabinoids including ethanol, butane, propane, hexane, petroleum ether, methyl tertbutyl ether, diethyl ether, carbon dioxide (CO_2_), and olive oil (Dussy et al. 2005; Lehmann and Brenneisen 1992; Romano and Hazekamp 2013; Rovetto and Aieta 2017). Gaseous solvents such as butane and propane can also be used for extraction purposes (Raber et al. 2015). Gas solvent extractions start in the gas phase at room temperature and are either cooled or pressurized into a liquid state as they run through the sample material (Rovetto and Aieta 2017). The extracted sample is collected, and the solvent is evaporated (Chan et al. 2017). The process of pressurizing these flammable and potentially explosive gases poses safety hazards (Jensen et al. 2015). In addition, the gases used in cannabis extractions are often industrial grade and contain impurities that end up in the cannabis extracts. Moreover, the solvents themselves may become a residue in the final extract (Raber et al. 2015).

The differing solubilities of individual cannabinoids and other phytochemicals are thought to be an important factor that needs to be considered when selecting a solvent. The stickiness and viscosity of cannabis oil result in binding to solvents; therefore, it is important to consider the toxicity, affinity, and temperature profile of the solvents being used (Fathordoobady et al. 2019). The efficiency of conventional methods of extraction is presented to be heavily dependent on the solvent of choice. Solubility, molecular affinity, mass transfer, co-solvent, toxicity, and environmental safety are major factors that should also be considered during the solvent selection process (Azmir et al. 2013). Commonly used solvents to extract cannabis can be divided into three groups, low molecular mass organic solvents, vegetable fats (oils), and supercritical fluids, notably supercritical carbon dioxide (Reichardt and Welton 2011).

#### Low molecular mass organic solvents

Low molecular mass organic solvents are hydrocarbon-based with limited polarity due to the presence of oxygen. Halogen substituted hydrocarbons are also included in this group.

These solvents are known for their ability to dissolve generally nonpolar compounds, following the chemistry adage: like dissolves like. Inspection of cannabinoids in Table 2 shows that they are dominated by carbon and hydrogen, making them generally nonpolar. However, the presence of alcohol and acid groups requires some polarity in extraction solvents and solvent mixtures.

**Table 2:** Popular low molecular mass organic solvents

Name	Formula	Polarity	Molar mass (g mol^−1^)	Boiling point (°C)
Ethanol	C_2_H_5_OH	Polar	46.07	78.4
Butane	C_4_H_10_	Nonpolar	58.12	− 1.0
Hexane	C_6_H_14_	Nonpolar	86.18	68.0
Methanol	CH_3_OH	Polar	32.04	64.7
Acetone	C_3_H_6_O	Polar	58.08	56.0

Table 2 shows some of the properties of the most popular organic solvents in cannabis extraction. Notably absent from this popular group are dichloromethane and chloroform, both halogenated hydrocarbons are commonly used in analytical fat/oil extraction from plant and animal tissue. These solvents are observed to have low boiling points and high volatility, indicating their ability to be easily separated from the extract at low temperatures after the extraction process (Reichardt and Welton 2011).

To illustrate how different solvents can affect the yield of compounds from the source material, consider the example of phenolic extraction from grape pomace and elderberry. Phenols are nominally water soluble. The solvent combinations ethanol–water and acetone–water mixtures had a higher yield than ethyl acetate-water mixture (Vatai et al. 2009). In another example, isopropanol-hexane, chloroform–methanol, and hexane were used as solvents for crude fat extraction from insect, egg yolk, and krill powders in one-step organic solvent extraction. The highest fat yield was achieved with a chloroform–methanol mixture (Rose 2019). Thus, with a mixture of cannabinoids, terpenes, chlorophyll, carotenoids, and other fat-soluble classes in cannabis flowers, different extraction efficiencies can be confidently predicted. If seeds have matured, the fats (triacylglycerols) that comprise the energy stored in seeds will also be extractable to some extent.

Namdar et al. (2018) reported that for cannabis plant extraction, the ratio and the nature of the solvents can determine the evaporation time after extraction, which should be minimized. A mixture of polar and non-polar solvents achieved the highest yield for all the compounds in the cannabis plant (Namdar et al. 2018).

#### Vegetable fats (oils)

**Fig. 2:**
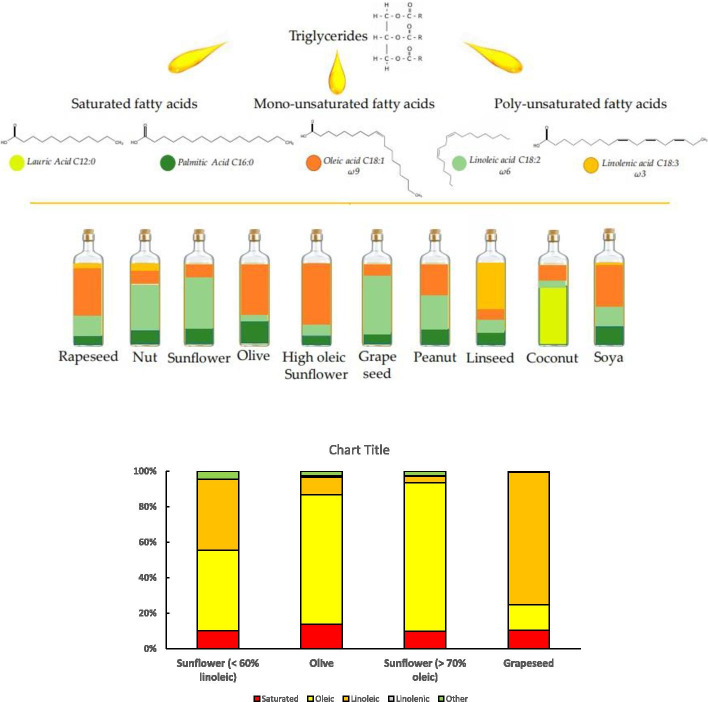
Vegetable oils composition by fatty acid profile, inspired by Yara-Varón et al. (2017)

Vegetable oils are routinely extracted from seeds or fruits such as rapeseed, sunflower, or olive, and even brans, making them an inexpensive option. These oils are considered lipophilic due to their nonpolar characteristic, which enables selective dissolving properties. Approximately, 95 to 98% of vegetable oils consist of triglycerols whose composition is dominated by six fatty acids (Yara-Varón et al. 2017). Figure 2 shows the major fatty acids in different vegetable oils (Yara-Varón et al. 2017). Each of these has a degree of emulsifying capacity that may play a role in cannabinoid extraction. Interestingly, apart from olive oil, some specialized oils, nearly all commercial oils, are refined to eliminate the minor components. Whether this could affect cannabinoid extraction is unknown.

Olive oil is a well-known solvent in the cannabis extraction field. It is also one of the least refined oils with characteristically high oleic acid content. Terpenes can be preserved during extraction with olive oil due to their low volatile nature. Romano and Hazekamp (2013) used two different protocols with olive oil for cannabis extraction. In the first experiment, 5 g cannabis with 20 ml olive oil and 50 ml water were mixed and heated up to 60 min. In the second experiment, 10 g cannabis with 100 ml olive oil were mixed and heated for up to 120 min. The extract concentration to the solvent ratio for the first and second protocols was 5 g/20 ml and 10 g/100 ml, respectively. The high yield of terpenes obtained from using olive oil as a solvent is thought to be due to its efficient capabilities in solubilizing and limiting loss of product by protecting the compounds from evaporation (Romano and Hazekamp 2013).

#### Supercritical carbon dioxide (CO_2_)

**Fig. 3:**
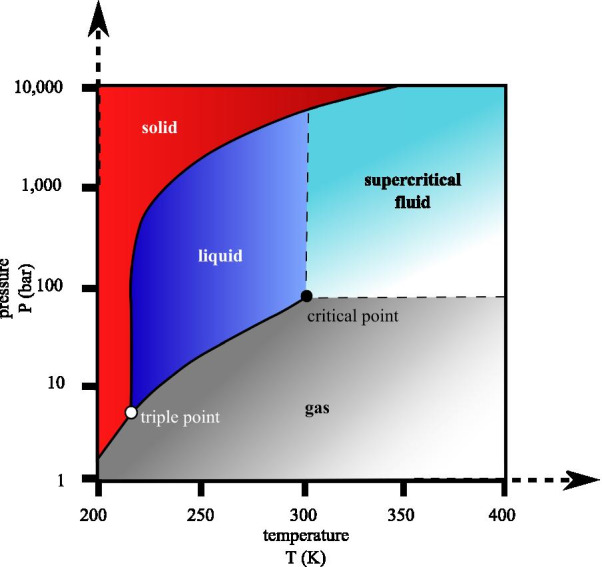
CO_2_ pressure–temperature phase diagram, the critical temperature is 304.13 K or 31.0 °C or 87.8°F, and the critical pressure is 7.3773 MPa or 72.8 atm or 1070 psi or 73.8 bar. (Adopted from Wikimedia commons URL: https://upload.wikimedia.org/wikipedia/commons/1/13/Carbon_dioxide_pressure-temperature_phase_diagram.svg)

In common with other solvents, CO_2_—which is nominally a polar gas—enters a so-called supercritical state at a defined temperature and pressure. In a supercritical state, distinct liquid and gas phases do not exist. In the case of CO_2_, the critical temperature is 31.06 °C, the critical pressure is 73.83 bar, and the critical density is 0.460 g/cm^3^ (Raventós et al. 2002). Supercritical CO_2_ behaves like a non-polar solvent, capable of extracting a broad range of non-polar solutes, cannabinoids included. In comparison, strongly polar water becomes supercritical and useful as a non-polar solvent but at a much higher temperature and pressure, 647 K and 22.1 MPa (Fig. 3). Therefore, CO_2_ is the solvent of choice due to low critical temperature and pressure. It is also non-flammable, non-toxic, inert, renewable, easy to remove, abundant, and relatively low-cost. As an example, consider supercritical extraction of linalyl acetate from lavender oil compared with its extraction by conventional steam distillation (Reverchon et al. 1995). The yields for supercritical extraction were 34.7% compared with 12.1% for the conventional steam distillation. The reason proposed was that the higher temperature of steam distillation caused the undesirable hydrolysis of the linalyl acetate to linalool and acetic acid.

Thus, the low base temperature of supercritical CO_2_ is probably an intrinsic advantage (Reverchon et al. 1995).

### Conventional methods of extraction

#### Soxhlet extraction

Soxhlet extraction was first proposed by Franz Ritter Von Soxhlet, a German chemist, as a method of extraction of, primarily, lipids. However, over the years, this procedure has become widely employed for various extraction purposes, commonly used for the separation of bioactive compounds from plant matter. Soxhlet is also extensively used as a model for the comparison and development of alternative methods of separation (Azmir et al. 2013). The process begins by placing a small amount of the dried sample in a thimble that is then transferred to a distillation flask containing a particular solvent. When the overflow level is reached by the solution, a siphon is used to aspirate the solute and unload it into the distillation flask with the extracted analyte carried along into the bulk liquid. This procedure is repeated several times until total extraction is complete (Luque de Castro and García-Ayuso 1998). For cannabis extractions using the Soxhlet apparatus, Lewis-Bakker et al. (2019) compared different types of organic solvents for the procedure and found ethanol had exhibited the highest yields of cannabinoids (Lewis-Bakker et al. 2019). As commonly witnessed by other conventional processes, the long-running time and the large amount of solvent required are limitations that not only increase the cost of operation but also cause environmental complications (Luque de Castro and García-Ayuso 1998). These drawbacks were demonstrated by a study conducted by Wianowska et al. (2015) that compared the extraction profiles of THCA and THC using the Soxhlet extraction procedure. It was clear that the long-lasting high temperature accentuated the degradation pathway from THCA to THC and finally to CBN, resulting in high levels of THC and CBN (Wianowska et al. 2015).

The simplicity in methodology alongside the ease of system optimization can result in high sample throughput and yield. The minimal requirement for a trained personal for process operation is also considered advantageous when compared to recently developed methods of extraction. Soxhlet methods can be manual or automatic, and the latter is less hazardous and allows multiple treatments to be examined simultaneously to optimize solvent composition, solvent to plant ratio, and extraction time (Luque de Castro and García-Ayuso 1998).

#### Dynamic maceration (DM)

Dynamic maceration is a conventional solid-lipid extraction procedure that is based on soaking a sample in organic solvents (solvent varies depending on the polarity of the target compound) for a specific time at a specific temperature and followed by agitation (Fathordoobady et al. 2019). This process of separation is inexpensive and a popular method used to obtain essential oils and bioactive compounds (Azmir et al. 2013). Recently, the use of vegetable oils (e.g., olive oil) as maceration extraction solvents was found to be more useful for extracting higher amounts of terpenes than alcoholic solvents, notably when using extended heating time. However, vegetable oils are not volatile and are difficult to remove from extracted isolates (Romano and Hazekamp 2013). Alternatively, ethanol is suggested as a preferred solvent for cannabinoid extraction. A study conducted by Fathordoobady et al. (2019) demonstrated that there was no significant difference between other organic solvents (n-hexane, acetone, methanol) and ethanol when used for neutral cannabinoid recovery. However, when the recovery of acidic cannabinoids was tested, ethanol had the highest yield. The use of ethanol for maceration extraction of cannabinoids was found to produce the highest yield when used twice compared to other methods of extractions, for instance, ultrasonic-assisted extraction (UAE) or supercritical fluid extraction (SFE) (Fathordoobady et al. 2019).

Romano and Hazekamp (2013) compared five different solvents (naphtha, petroleum ether, ethanol, olive oil + water, and olive oil) using DM (Table 3). Except for naphtha, other extracts contained a small amount of THC and THCA around 5–10%. Naphtha was an exception which had 33% THC plus THCA. With ethanol as solvent, unwanted chlorophyll was extracted along with the cannabinoids. The unwanted chlorophyll not only added an unpleasant flavor and a green tinge to the end product, but it also demonstrated accounts of interference with gas chromatography–mass spectrometry analysis, hence removal is considered necessary (Ciolino et al. 2018). To eliminate unwanted chlorophyll, the ethanol extract can be treated with activated charcoal. However, the use of activated charcoal can result in the reduction of cannabinoid content by approximately 50%. Consequently, although yields are high with ethanol, the removal of unwanted chlorophyll with charcoal comes at the expense of cannabinoid loss. In respect of toxicity, Romano and Hazekamp (2013) found significant amounts of petroleum hydrocarbon residues in the extracts obtained with naphtha and petroleum ether, indicating that special attention must be paid to ensure safe residual concentrations (Romano and Hazekamp 2013).

**Table 3:** Five protocols to extract cannabis by dynamic maceration (Romano and Hazekamp 2013)

Cannabis (g)	5	5	5	5	10
Solvent (mL)	Naphtha (200)	Petroleum ether (200)	Ethanol (200)	Olive oil (20) + water (70)	Olive oil (100)
Extraction/filtration 1	5 g Cannabis + 100 mL solvent, agitate 20 min	5 g Cannabis + 100 mL solvent, agitate 20 min	5 g Cannabis + 100 mL solvent, agitate 20 min	5 g Cannabis + 20 mL olive oil + 50 mL water; heat at 100 °C 60 min	10 g cannabis + 100 mL olive oil; heat at 100 °C 120 min
				Cool	Cool
	Paper filtration	Paper filtration	Paper filtration	Paper filtration with pressure	Paper filtration with pressure
Extraction/filtration 2	100 mL solvent, agitate 20 min	100 mL solvent, agitate 20 min	100 mL solvent, agitate 20 min	20 mL of hot water	
	Paper filtration	Paper filtration	Paper filtration	Paper filtration with pressure	-
	Combine extracts	Combine extracts	Combine extracts	Combine extracts	
Extract clean up	None	None	Optional activated charcoal filtration	None	None
Evaporation/separation	Boiling water bath with nitrogen stream	Boiling water bath with nitrogen stream	Boiling water bath with nitrogen stream	Phase separation; freeze; decant oil phase	None
Reconstitution	Reconstitute residue with ethanol to 100 mL	Reconstitute residue with ethanol to 100 mL	Reconstitute residue with ethanol to 100 mL		Collect the oil
Extract concentration (cannabis/solvent)	5 g/100 mL	5 g/100 mL	5 g/100 mL	5 g/20 mL	10 g/100 mL
Dilution factor for analysis	20 ×	20 ×	20 ×	100 ×	40 ×
Final concentration (cannabis/solvent)	2.5 mg/mL	2.5 mg/mL	2.5 mg/mL	2.5 mg/mL	2.5 mg/mL

In the same study, when compared to other solvents, the olive oil extract was shown to contain the largest number of terpenes, making it a superior crude extract. Olive oil is a cost-effective nonflammable solvent that is considered nontoxic when applied topically or consumed orally, and not through the lungs. As an added benefit, Citti et al. (2016) recognized that olive oil-based cannabis extracts maintained their cannabinoid concentration longer than ethanol-based extracts. A disadvantage associated with olive oil extracts, however, is that extracts cannot be concentrated by evaporation. This means that larger volumes of olive oil extracts need to be consumed to have the same therapeutic effects as other extracts (Romano and Hazekamp 2013). In another study by Hazekamp et al. (2009), hexane—the usual form of petroleum ether—was used as a solvent for the maceration method in fiber and drug varieties of cannabis. The yields of cannabinoids were discovered to be 3% and 17%, respectively. For this study, hexane was particularly used as it does not extract chlorophyll and is easily evaporated after extraction (Hazekamp et al. 2009).

Methods to extract chlorophyll from plants generally required acetone as the preferred solvent; however, as acetone is considered carcinogenic, it is not recommended to be used in cannabinoid extraction. Namdar et al. (2018) extracted cannabinoids with ethanol (partly polar) and hexane (non-polar), and their mixture. The highest yield was achieved with the mixture, but for cannabinoids, the polar solvent was best (Namdar et al. 2018). Likewise, Brighenti et al. (2017) concluded that dynamic maceration with ethanol for 45 min at ambient temperature was the best way of extracting non-psychoactive cannabinoids especially the acidic forms compared to more elaborate methods like ultrasonic-assisted extraction (UAE) (Brighenti et al. 2017).

### Alternative methods of extraction

#### Ultrasonic-assisted extraction (UAE)

Ultrasound technology is widely adopted in the food and chemical industry for its ability to significantly influence the rate of various processes (Chemat et al. 2008). The main feature that sets ultrasonic-assisted extraction (UAE) apart from other processes is the use of sound waves, commonly with frequencies between 20 to 100 kHz. This enables the penetration of solvents into a sample matrix to extract the compounds of interest. This is done during the process of cavitation. Cavitation is described as the formation, expansion, and collapse of bubbles within the solution that allows for intense mass transfer and accelerated solvent access into cell material (Azmir et al. 2013). The effective mixing ability of the UAE can be explained by the faster energy transfer, micro-mixing, and reduced extraction temperature (Otles 2016). Factors such as moisture content of a sample, particle size, milling degree, solvent, temperature, pressure, and time of sonication must be considered and manipulated to achieve efficient extractions (Azmir et al. 2013). A study that employed the ultrasonication method to leach and hydrolyze phenolic compounds presented evidence of low analyte decomposition during the extraction procedure when compared to other methods such as subcritical water, and microwave-assisted and solid–liquid extractions. After assessing the degradation of phenolic compounds, the decrease in decomposition was found to be due to the low energy type produced by the sonication mechanism and the short duration time. However, this was only evident when the exposure time to ultrasound was less than 10 min (Herrera et al.^ 2005^).

De Vita et al. (2018) compared different methods for the extraction of commercially available hemp and medicinal cannabis to evaluate the changes in cannabinoid composition. The experimentation demonstrated the optimal conditions for the highest yield of cannabinoids using ultrasonication to be 50 min at 60 °C with ethanol as a solvent. Despite the optimal conditions, the total amounts of THC and CBD extracted were slightly lower when compared to the controls, which were obtained under reflux at 90 °C for 50 min in ethanol. Although low yield was obtained, the ultrasonication procedure had provided extracts using lower temperatures in an environmentally friendly, safe, and energy-efficient way. This study also found that ethanol extract yield was 3 to 4 times higher than olive oil extract (De Vita et al. 2020). To further explore the concept of solvent influence in UAE, Lewis-Bakker et al. (2019) conducted an extraction procedure with the following parameters: UAE in 80 W of ultrasonic bath power, 63 W of heating power, at 40 kHz for 5 min. A mix of ethanol, hexane, and isopropanol: hexanes (1:1) were used as solvents. The results showed that the yield for ethanol and hexane was almost the same, and isopropanol: hexanes achieved the highest yield of the extract. However, an HPLC analysis showed a reverse relationship between the extract yield and cannabinoids: the isopropanol: hexanes product had the lowest cannabinoid content, due to coextracted non-cannabinoid content. The authors also indicated that the acidic forms of cannabinoids (four shown in Fig. 2) were almost intact with UAE extraction compared to other methods (Lewis-Bakker et al. 2019). To optimize the extraction of target cannabis compounds, it is suggested to use UAE as a conditioning step for conventional extraction methods. For example, it was found that using UAE before a Soxhlet extraction improved the crude lipid yield by more than 24% without affecting the quality of extract (Fathordoobady et al. 2019).

#### Microwave-assisted extraction (MAE)

**Fig. 4:**
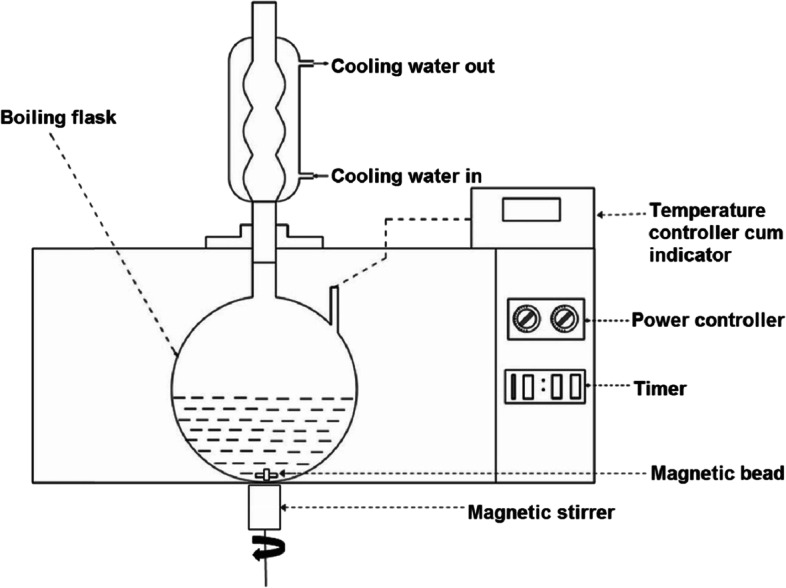
MAE process where the flask is housed in the microwave oven (Krishnan and Rajan 2017). Placing the flask containing the sample in the microwave, attached to a condenser outside of microwave to capture the solution of interest compounds after distillation

In 1980, the increasing demand for environmentally friendly and sustainable industrial processes had provoked the development of the Microwave-assisted extraction procedure (Otles 2016). The electromagnetic energy provided in the form of microwaves, with frequencies between 300 MHz and 300 GHz, is used to produce rapid heating following ionic conduction and dipole rotation (Azmir et al. 2013). This procedure directly exposes each molecule to a microwave field which is converted to kinetic energy that can break cell walls and release their contents into a liquid phase. The enhanced performance of this green extraction process can be attributed to improved solubility, efficient mass transfer, and increased surface equilibrium. These factors result in a system that uses less energy with fast processes requiring less solvent consumption but also producing a final product with high purity (Fig. 4) (Ani et al. 2012). De Vita et al. (2018) used MAE to explore time, temperature, ramping time, and solvent as variables. The study demonstrated that the extraction yield of CBD increased with increasing temperature and duration by at least 4 times when compared to the reference sample, which was prepared by ethanol reflux at 90 °C for 50 min. It was also noted that olive oil had superior properties when compared to ethanol during an MAE (De Vita et al. 2020).

Neutral phytocannabinoids have been established as important for their medicinal properties; therefore, using extraction procedures to obtain these compounds is considered essential. Methods used for the extraction of neutral cannabinoids can be explored by investigating their decarboxylation efficiencies of phytocannabinoid acids. For example, Lewis-Bakker et al. (2019) had studied the processes of different isolation methods and found MAE to be superior in terms of yielding high neutral cannabinoids. The study had found high temperature (> 130 °C) led to decarboxylation of more than 99% of acidic cannabinoids during MAE. To further promote the decarboxylation of acidic phytocannabinoids, MAE was used for 10 min at 150 °C with extracts from prior Soxhlet, UAE, and SFE extractions. However, only the isolates from the Soxhlet method had completely decarboxylated. Although prolonging the duration time to 30 min in MAE, extracts yielded 0.6% CBN. As CBN is produced from the oxidation changes of THC, this can be due to a radical-mediated or oxidation during MAE (Lewis-Bakker et al. 2019).

#### Pressurized liquid extraction (PLE)

**Fig. 5:**
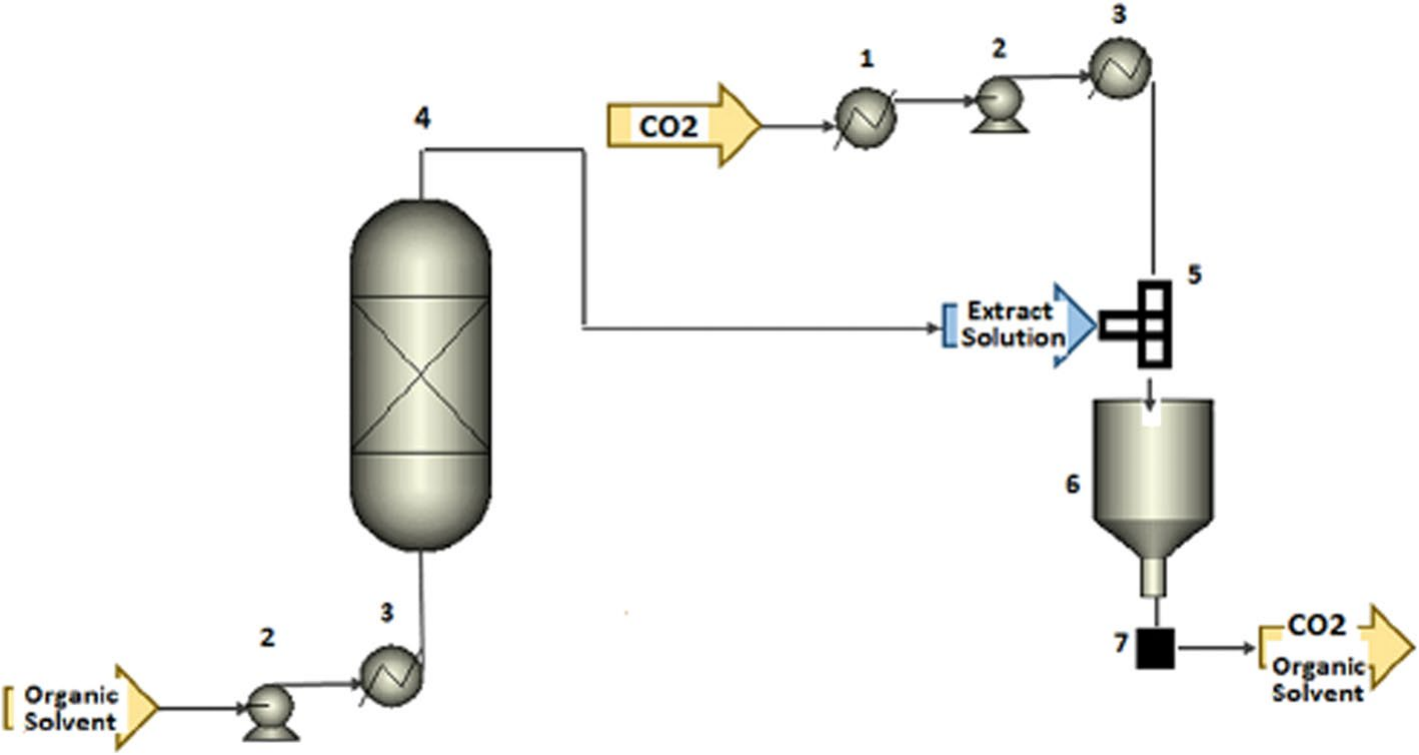
PLE process using organic solvent as extracting solvent coupled with supercritical antisolvent (SAS) precipitation process (1) heat exchanger for cooling, (2) pump, (3) heat exchanger for heating, (4) extractor, (5) T-mixer, (6) precipitation vessels, and (7) filter (Santos and Meireles 2015)

Pressurized liquid extraction (PLE), also known as accelerated solvent extraction (ASE) (Duarte et al. 2014), is documented to be a highly efficient and rapid method of compound extraction. In this approach, high pressures facilitate the extraction while the high temperatures promote solubility and mass transfer to increase analyte solubility, as well as reduce solvent viscosity and surface tension (Azmir et al. 2013). Accordingly, altering temperature and pressure enables influence over the solubility of the compound of interest (Wianowska et al. 2015). This procedure also does not require a filtration step as the insoluble matrix components are contained inside the extraction cell. This feature allows for the process automation for continuous operation (Fathordoobady et al. 2019). Figure 5 visualizes the PLE process.

When comparing PLE to conventional methods such as Soxhlet, features such as shorter duration, reduced solvent consumption, and decreased sample handling are observed (Rodrigues et al. 2016). To demonstrate this, Wianowska et al. (2015) compared the amount of THCA, THC, and CBN obtained from a Soxhlet and PLE process with two types of extractants, methanol, and n-hexane. Employing methanol as an extractant, the first set of results had indicated, even in high temperatures, the concentration of THC was lower than THCA using the PLE method. The Soxhlet process had contrasting results as the concentration of THC was much higher than THCA. The data obtained illustrates the influence of parameters such as time and pressure have on the end product. The high pressure applied enables the use of temperatures above the boiling point of the extractant. This increases the penetration ability of the selected solvent into the plant matrix in a short time. The high temperature used in PLE does not avoid the transformation of THCA and THC to CBN; however, the degree at which this occurs is found to be much lower than that demonstrated by the Soxhlet extraction (Wianowska et al. 2015).

For the extraction of cannabis constituents, Fathordoobady (2019) demonstrated that by using methanol and acetone/methanol (50:50) as solvents with PLE parameters of 1250 bar at 60 °C temperature, 17 various compounds, and three cannabinoids (Δ9-THC and its metabolites 11-nor-9-carboxy-THC and 11-hydroxy-THC) were identified from the cannabis plant (Fathordoobady et al. 2019).

#### Supercritical fluid extraction

Green approaches, such as supercritical fluid extraction (SFE), are used to displace conventional methods of pressing and organic solvent extractions. These procedures decrease environmental impacts and reduce toxic residue on products by using supercritical fluids (Aladić et al. 2015). The process behind SFE can be condensed into two steps: (1) the plant material of interest is solubilized in a supercritical solvent of choice, commonly CO + , to extract the desired compound. (2) Those compounds are then recovered from the solvent to produce the end product. The use of supercritical fluids is advantageous as at room temperature they are in a gaseous, allowing for recovery of extract via simple evaporation (Santos and Meireles 2015). The differing solubilities of different solvents allow for selective extraction, as small variations to pressure and/or temperature can allow for selectivity (Perrotin-Brunel 2011). The employment of low temperatures is also considered advantageous as it results in low energy consumption as well as allowing for the preservation of thermosensitive compounds, such as cannabinoids (Aladić et al. 2015).

**Fig. 6:**
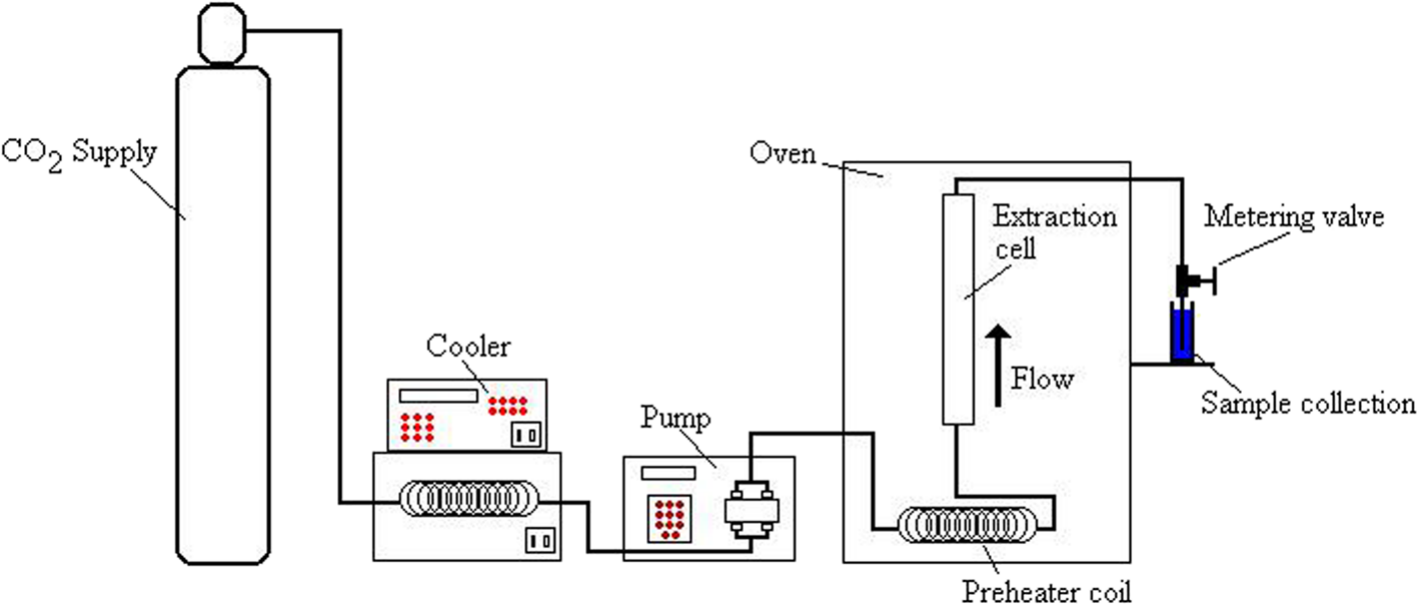
Diagram of a supercritical fluid extraction (Adopted from Wikiwand.com URL: https://www.wikiwand.com/en/Supercritical_fluid_extraction#)

Under conditions except for supercritical, CO_2_ behaves as a polar compound. In instances where supercritical CO_2_ is not sufficiently polar to act as a solvent, polarity modifiers, such as alcohols, water, and acids, can be used as co-solvents (Rovetto and Aieta 2017). However, CBD and THC are soluble in supercritical CO_2_ because they are dominantly nonpolar, making this the solvent of an appropriate choice (Grijó et al. 2018). Rovetto and Aieta (2017) evaluated the effect of pressure and the use of ethanol as a co-solvent on cannabinoid extraction. Extractions were run at 17, 24, and 34 MPa pressure. The yields increased almost linearly to 34 MPa, 0.185 g/g of cannabis at this pressure, compared with yield from a traditional ethanol extraction of 0.132 g/g. Increased pressure can increase the solvation power but decreases the selectivity of the extraction, so a higher pressure may not be the ideal condition. Ethanol was indicated to be useful as a co-solvent: When added in pulses, it can increase the rate of supercritical CO_2_ extraction of cannabinoids (Rovetto and Aieta 2017). Omar et al. (2013) also demonstrated that using a co-solvent can increase the yield (Omar et al. 2013). The optimum yield of these cannabinoids was achieved by using ethanol as co-solvent at 55 °C and 34 MPa (Fathordoobady et al. 2019). However, when comparing SFE with other methods of extraction, Brighenti et al. (2017) revealed that the lowest amount of CBDA, CBD, and CBG was obtained (Brighenti et al. 2017). Figure 6 visualizes the supercritical fluid extraction process.

#### Hydrodynamic cannabis extraction

Hydrodynamic cannabis extraction is a recent development within the cannabis industry that can be used to produce full-spectrum cannabis extracts with high bioavailability. There have been accounts of companies, such as IASO (Incline Village, Nevada), claiming to have developed a unique extraction system that produces products with high yield and increased potency. This alternative method involves freezing fresh plant material and converting it into a nanoemulsion in water by ultrasonication. Hydrodynamic force is then used to break the cell wall and release its contents. This is followed by liquid–liquid extraction using solvents, centrifugal separation, and finally low-temperature drying. The initial step of freezing the plant matter helps preserve the volatile compounds as well as acidic cannabinoids during the following steps. Hydrodynamic extraction is claimed to exceed conventional methods mainly due to the lack of high temperatures, short contact distillation, and low organic solvent consumption (admin, n.d.). Ishida and Chapman (2012) used this technique to extract carotenoids from tomatoes and found that the extractable lycopene, other carotenoids, and accessibility of carotenoids significantly improved (Ishida and Chapman 2012). However, to this date, there has been no scientific publication that explores this method of extraction. Therefore, to fully understand the efficacy of this method, more research is required.

## Discussion

Traditionally, the dried cannabis flower was the product of choice; however, as the industry expands, the demand for various products with distinct properties also increases. Therefore, multiple factors should be considered when selecting a drying technique or an extraction method to produce a specific product. Among different drying methods for post-harvest processing, freeze-drying is considered more appropriate when compared to other methods; however, there is currently a lack of academic research and evidence to support this. Hang-drying as a traditional technique is still the most convenient way to reduce the prevalence of mold and bacteria during storage before extraction. Solventless extraction and hydrodynamic extraction are of interest due to their high yield, easy, and fast process but lack the scientific publication to promote their employment for large-scale production. According to cannabinoids’ lipophilic or hydrophobic properties, slightly polar solvents are recommended for extraction. Although for terpenes with more than 15 carbons, non-polar solvents are suggested. Soxhlet and dynamic maceration are being used as traditional methods which are time- and solvent-consuming but accurate enough to be compared with modern techniques. Among modern methods, SFE, MAE, and UAE are well recognized as feasible and convenient techniques.

## Conclusion

In this narrative review paper, the advantages and disadvantages of various drying and extraction methods have been discussed. The best methods for industries based on the final products have been reviewed and suggested. Some gaps are found in this review paper including the lack of information and knowledge about using freeze dryer for drying plant material after harvest, hydrodynamic extraction method, and a developed green extraction technique in the cannabis research area as well as cannabis industry which needs more investigations in the future studies.

## Data Availability

Data sharing is not applicable to this article as no datasets were generated or analyzed during the current study.

## Abbreviations

THC: (-)-Trans-Δ^9^-tetrahydrocannabinol
THCA: (-)-Trans-Δ^9^-tetrahydrocannabinolic acid A
∆9-THC: (-)-Trans-Δ^9^-tetrahydrocannabinol
∆8-THC: (-)-Trans-Δ^8^-tetrahydrocannabinol
CBD: Cannabidiol
CBDA: Cannabidiolic acid
CBG: Cannabigerol
CBGA: Cannabigerolic acid
CBC: Cannabichromene
CBCA: Cannabichromenic acid
CBN: Cannabinol
ECS: Endocannabinoid system
UAE: Ultrasound-assisted extraction
MAE: Microwave-assisted extraction
DM: Dynamic maceration
SFE: Supercritical fluid extraction
HPLC: High-performance liquid chromatography
PLE: Pressurized liquid extraction
